# A Rational Approach to Unilateral Neck RT for Head and Neck Cancers in the Era of Immunotherapy

**DOI:** 10.3390/cancers13215269

**Published:** 2021-10-20

**Authors:** Re-I Chin, Joshua P. Schiff, Randall J. Brenneman, Hiram A. Gay, Wade L. Thorstad, Alexander J. Lin

**Affiliations:** Department of Radiation Oncology, Washington University School of Medicine, Saint Louis, MI 63110, USA; rchin@wustl.edu (R.-I.C.); j.p.schiff@wustl.edu (J.P.S.); rjbrenneman@wustl.edu (R.J.B.); hiramgay@wustl.edu (H.A.G.); thorstad@wustl.edu (W.L.T.)

**Keywords:** radiation therapy, head and neck cancer, immunotherapy, unilateral neck RT

## Abstract

**Simple Summary:**

Elective radiation of clinically uninvolved lymph nodes for head and neck cancer should balance the risk of treating occult metastatic disease and maintaining patient quality of life. Clinical trials exploring combining radiation with immunotherapy have thus far been disappointing. This review proposes limiting elective neck radiation to reduce iatrogenic immunosuppression in future trials of immunoradiation.

**Abstract:**

Radiotherapy plays an important role in the definitive and adjuvant treatment of head and neck squamous cell carcinoma (HNSCC). However, standard courses of radiation therapy may contribute to the depletion of circulating lymphocytes and potentially attenuate optimal tumor antigen presentation that may be detrimental to the efficacy of novel immunotherapeutic agents. This review explores the advantages of restricting radiation to the primary tumor/tumor bed and ipsilateral elective neck as it pertains to the evolving field of immunotherapy.

## 1. Introduction

Head and neck squamous cell carcinoma (HNSCC) is a common malignancy accounting for over 65,000 new cases and 14,000 deaths in the United States in 2020 [1], and the incidence of late-stage head and neck cancer has been rising [2]. Locoregionally advanced (stage III/IV) HNSCC is associated with a high risk of both locoregional relapse and distant metastases, often requiring combined modality approaches involving surgery, radiation therapy (RT), and/or chemotherapy to maximize long-term disease control [3,4,5].

For metastatic HNSCC, immunotherapy with or without the addition of chemotherapy represents a standard of care treatment option [6,7,8]. These immunotherapy agents include monoclonal antibodies (mAb) targeting the programmed death protein/ligand 1 (PD-1, PD-L1) and cytotoxic T lymphocyte antigen-4 (CTLA-4) immune checkpoint signaling axes [9]. Despite preclinical data suggesting synergy between radiotherapy and immunotherapy [10,11,12], recent data on the addition of the PD-L1 mAb avelumab to standard of care chemoradiation for locally advanced HNSCC have been disappointing [13]. In the metastatic setting, a phase II trial evaluating the anti PD-1 mAb nivolumab with or without stereotactic body radiotherapy (SBRT) found combination therapy did not improve the overall response rate (ORR) (22% vs. 26.9%, *p* = 0.94), progression-free survival (PFS) (2.4 months vs. 1.9 months, *p* = 0.8), or overall survival (OS) (53% vs. 64%, *p* = 0.79) at one year [14]. Ongoing clinical studies on immunotherapy and radiotherapy in head and neck cancers are provided in Table 1. Given the historically suboptimal response rates to checkpoint blockade across most cancer sites, careful patient selection based on histological and immunological factors is needed to fully realize the potential of immunotherapy in the setting of locally advanced head and neck cancer.

RT can inhibit generation of anti-tumor immune responses by either directly killing radiation-sensitive circulating lymphocytes or via antagonism of antigen presentation in draining lymph nodes [27]. Eliminating RT to the elective contralateral neck has been shown to reduce the severity of radiotherapy-induced lymphopenia compared to bilateral neck RT, an effect likely attributable to reducing total irradiated blood volume [28,29,30]. Yet outside select cases of lateralized tonsil [31,32,33], salivary gland [34], or buccal mucosal cancers [35], routine implementation of unilateral neck RT is limited due to concerns regarding patterns of micrometastatic lymph node spread in more midline primary tumors [36].

This review aims to provide a rational overview of the clinical approach to unilateral neck radiotherapy for anticipated synergy with systemic immunotherapeutic approaches. We explore current data as well as future directions for immunotherapy, patient selection for unilateral radiotherapy, and ongoing clinical studies that incorporate immunotherapy with or without unilateral neck RT.

## 2. Materials and Methods

We searched PubMed and ClinicalTrials.gov from the date of their inception until 1 August 2021, for relevant articles using the following key terms: “head and neck cancer” AND “radiation therapy” AND “immunotherapy” AND “unilateral neck treatment.” All relevant keyword variations were used for these terms, and we restricted our searches to reports published in English.

## 3. Preclinical and Clinical Data Combining Immunotherapy and RT in HNSCC

As is the case with most solid tumors, the tumor microenvironment appears to play a critical role in the development of HNSCC. Ligands for inhibitory receptors on circulating lymphocytes, such as CTLA-4, PD-1, (PD-L1 is the ligand for PD-1) and T cell immunoglobulin mucin-3 (TIM-3), are overexpressed by HNSCC tumor cells and are associated with worse survival [37,38,39]. Inhibiting lymphocyte effector function impairs their ability to be recruited to and traverse the tumor microenvironment. Conversely, high CD8^+^ tumor infiltrating lymphocyte (TIL) counts were associated with an improved 3-year overall survival (OS) in patients with human papillomavirus (HPV)-positive oropharyngeal cancer [40].

Most preclinical data supporting the synergy between radiotherapy and immunotherapy utilizes hypofractionated courses of RT, generally 1 to 5 doses of 5–10 Gy per dose, targeting the clinically apparent tumor. Compared to a single modality treatment, this approach has been shown to improve tumor control via a variety of mechanisms, including immune priming, release of tumor-specific antigens, and generation/maturation of CD8^+^ T cells in the tumor microenvironment [11,41,42,43]. Conventional fractionation schedules (1.6–2 Gy/day over 6–7 weeks of RT) used to treat HNSCC may skew the tumor microenvironment in favor of immunosuppression [44,45,46]. Morisada et al. demonstrated that conventionally fractionated RT, but not hypofractionated RT, reduced intratumoral CD8^+^ TIL effector activity, suppressed T-cell responses within tumor-draining lymph nodes, and mitigated the immune-enhancing effects of anti-PD-1 therapy in a syngeneic mouse oral carcinoma model [47].

Elective RT of draining neck lymph nodes could abrogate adequate antigen presentation and maturation of effector T lymphocytes. Marciscano et al. demonstrated that adding elective nodal RT led to a significant decrease in intratumoral CD8^+^ and CD4^+^CD25^+^FoxP3 regulatory T cells in murine models of transplantable colorectal carcinoma and melanoma [27]. Addition of elective nodal RT to immune checkpoint blockade using mAb and primary tumor RT decreased OS, mitigating the positive effects of anti-CTLA-4 mAb therapy [27]. In a syngeneic transplantable mouse model of breast cancer, use of pharmacologic agents to prevent T cells from exiting the tumor and draining the lymph nodes with radiotherapy led to the sequestration of primed CD8+ T cells [48]. These preclinical data suggest that conventionally fractionated RT and/or elective nodal irradiation may antagonize optimal tumor-directed immune responses, impairing immunotherapy efficacy.

The sequencing of RT with immunotherapy is also an area of active research [49]. Preclinical data suggests that anti-PD-1 therapy and radiotherapy are best given concurrently, as there may be significant synergy between these two therapies [50,51]. RT can trigger the release of immunogenic antigens, which synergize with checkpoint inhibitors to enhance the anti-tumor immune response via two proposed mechanisms. For one, neo-antigens released in response to RT may reinvigorate exhausted intratumoral CD8+ T-cells in conjunction with anti-PD-1 immunotherapy [50]. Alternatively, RT may stimulate the neo-antigen-induced proliferation and differentiation of naïve T-cells [50]. Together, these two mechanisms suggest that closely sequencing the checkpoint blockade with RT should be the goal in order to take advantage of the peak in-tumor effector T-cells.

The best clinical data regarding the sequence of RT and immunotherapy have come from the PACIFIC trial, where adjuvant durvalumab (anti-PD-L1) improved OS after definitive chemoradiation for locally advanced lung cancer [52,53]. Cancer cell lines which are resistant to the PD-1 blockade may require the priming of T cells prior to the initiation of immunotherapy [54]. In contrast, there are also data advocating for the use of radiotherapy after immunotherapy [55]. A study by Riaz et al. suggested that immunotherapy may decrease the tumor mutational burden over time in responders, in which case, radiotherapy may help consolidate treatment after checkpoint blockade therapy [55]. The sequencing of these therapies may also depend on the specific immunotherapeutic target [55]. A preclinical study by Young et al. evaluated the CTLA-4 blockade one week prior, one day after, and five days after radiotherapy and demonstrated the most significant benefit when the checkpoint blockade was initiated prior to radiotherapy [56]. Given the variability in these findings, further research evaluating the timing of the checkpoint blockade and radiotherapy is warranted.

## 4. RT Effects on Circulating Lymphocytes in HNSCC

Lymphocytes are among the most radiosensitive cells in the body [57], and even minimal total body radiation exposure can produce prolonged lymphopenia. In a commonly cited model, radiation-induced lymphopenia (RIL) severity correlates with the irradiated volume of blood pool/tissue and the number of daily treatments [30]. However, increasing the radiation dose rates from conventional (0.1 Gy/s) to FLASH levels (35 Gy/s) did not reduce RIL severity after 5 daily fractions of 1–2 Gy to the heart or spleen in a mouse model [58]. Preclinical evidence supports the existence of paracrine effects in RIL, which could be induced in non-irradiated mice through ex-vivo irradiation of blood followed by autologous reinjection [59]. A mouse model of glioblastoma suggested irradiation to the brain causes sequestration of lymphocytes in the bone marrow [60]. Therefore, the mechanism of RIL is not solely a physical function of irradiated blood volume but may include other changes to the blood milieu, causing prolonged lymphopenia.

Reduction in circulating lymphocytes potentially compromises the efficacy of immunotherapy [61,62,63]. In a study by Pike et al., patients treated with prolonged courses of palliative RT were more likely to develop severe RIL, and patients with severe RIL at the start of immunotherapy had increased mortality [62]. Chen et al. reviewed multiple phase I/II clinical trials and reported that RIL reduced the systemic disease control in patients treated with radioimmunotherapy, suggesting that RIL inhibited the immune responses in these patients [63]. RIL also predicted for a decreased OS in this study [63], suggesting that the compartment of circulating lymphocytes should be considered an organ at risk [64,65]. To estimate the dose to circulating blood cells from RT, investigators have developed a publicly available time-dependent computational framework, HEDOS (HEmatological DOSe), that evaluates the effect of different treatment plans, dose rates, and fractionation schemes on circulating blood cells [66].

Clinically significant RIL has also been observed in patients with HNSCC. Over 50% of patients with HNSCC treated at Johns Hopkins/Washington University with definitive or adjuvant radiotherapy developed severe grade 3–4 treatment-related lymphopenia, and this lymphopenia is associated with inferior OS in HPV-negative patients [28,44]. Observed rates of grade 3–4 lymphopenia were reduced when Washington University’s institutional policy changed to spare RT to the uninvolved contralateral neck (79% vs. 58%, *p* = 0.04) in patients with lateralized tonsil cancer, without compromising locoregional control rates [29,32]. In this retrospective review, more patients treated with unilateral neck RT received concurrent chemotherapy (86% vs. 69%), so the lymphopenia was attributed to the change in radiated blood volume [29].

The high rate of acute grade 3–4 lymphopenia seen with retrospective institutional data is discordant with what has been reported in prospective studies, perhaps due to differences in data availability or in the timing of the measurement of lymphopenia. In RTOG 0522, which evaluated the addition of cetuximab to chemoradiotherapy for locally advanced HNSCC, 4.3% of all patients (38/891) developed grade 3–4 lymphopenia [67]. In GORTEC 99-02, which evaluated various fractionation schemes for locally advanced HNSCC, grade 3–4 lymphopenia rates did not significantly differ between patients treated with conventional RT (11%) versus accelerated radiotherapy (14%) [68]. Additionally, in JAVELIN-100, which evaluated the addition of avelumab to chemoradiotherapy, grade 3–4 lymphopenia was observed in ~6% of patients in both the experimental and control groups [13]. Therefore, further validation of RIL in prospective studies should be pursued to determine the true rate of acute and chronic lymphopenia. Future trials should be designed to evaluate lymphocytes in the circulating blood pool, and bone marrow should be considered an organ at risk during RT concurrent with immunotherapy [64,65,69].

## 5. Refining the Double-Edged Sword of Neck Irradiation

In addition to improving response rates for patients receiving radiotherapy and immunotherapy, efforts to reduce the RT dose and elective treatment volumes for select patients with HNSCC may further improve QOL [32,70,71]. Studies reporting the Washington University experience on eliminating RT to the contralateral retropharyngeal and high level II lymph nodes in HNSCC [70] and contralateral elective neck in well-lateralized tonsil cancer [32] have demonstrated significant QOL improvement without compromising locoregional tumor control [32,70,71].

A phase II trial from Washington University demonstrated that omitting adjuvant RT to the contralateral neck for patients with pathologic node negative findings (pN0) after bilateral neck dissection maintained long-term function without compromising cancer control [71]. Despite the majority of patients (71%, 51/72) having primary tumors which crossed the midline, the non-irradiated neck control rate was 97% at a median follow-up of 53 months [71]. It is of note that this trial consisted of a highly selected patient population treated at an experienced, high-volume, comprehensive cancer center, which may contribute to the excellent outcomes observed. Global and physical quality of life measures returned to the baseline by 12 months after RT [71]. These results are currently being further tested in the multi-national phase three randomized PRESERVE trial (NCT03997643) [72].

Due to these initial results, our institutional treatment paradigm incudes utilizing bilateral neck dissections to potentially omit RT to the pN0 neck in patients with primary tumors approaching midline. Patients at Washington University with more midline HNSCC who have no clinical or radiographic evidence of contralateral nodal disease have the option of a staged contralateral neck dissection to assess for the pN0 status prior to the omission of contralateral neck radiotherapy. However, more prospective data from multiple institutions should be gathered before this concept should be widely adopted.

## 6. Controversies in Patient Selection for Unilateral Neck RT—Tonsil Cancer

The 2012 American College of Radiology Appropriateness Criteria recommends against unilateral neck RT for patients with N2b American Joint Commission on Cancer (AJCC) 7th edition disease [73]. The updated 2020 American Radium Society’s appropriate use criteria strongly recommends unilateral RT for a tonsil-confined tumor with ≤1 cm of tumor invasion into the soft palate or base of the tongue, and a minimal burden of nodal disease, such as 0–2 involved lymph nodes, for patients treated with definitive (chemo)radiotherapy [74]. However, the consensus on the appropriate use of bilateral neck irradiation in cases of multiple ipsilateral nodes, lymph nodes with clinical extranodal extension (ENE) or a single large (>6 cm) ipsilateral lymph node was not reached [74], so the RT approach for these patients remain controversial.

In the adjuvant setting, Rusthoven et al. delivered unilateral RT to 14 out of 20 patients with tonsil cancer with AJCC 7th pN1-N2b classification, with 80% of the patients having undergone surgery [75]. The majority of the patients had T1–2 (18/20) and N2b (13/20) disease, and unilateral treatment was only given for tumors without base of tongue or soft palate involvement, which resulted in no in-field or contralateral recurrences [75]. Similarly, Chin et al. reported the Washington University experience of 154 patients with palatine tonsil cancer treated with postoperative unilateral or bilateral IMRT [32]. For patients without bilateral neck disease, the primary selection criterion for unilateral RT was a well-lateralized primary tumor (>1 cm from midline) [76], regardless of ipsilateral pathological nodal stage, surgical margin status, or extranodal extension. The reported 5-year locoregional control rate was 97%, and did not significantly differ between ipsilateral versus bilateral elective nodal irradiation; 79% of patients treated with unilateral RT had stage AJCC 7th pN2a-b disease, 77% had extranodal extension, and no contralateral recurrences were observed [32].

A recent editorial [77] summarizes the published guidelines from several professional societies [73,74,78,79,80,81] and discusses the nuances of interpreting RT treatment policies involving AJCC 7th N2b and ENE as binary factors for patient selection for unilateral neck RT. Currently, the omission of contralateral RT is institution and context dependent, and can be influenced by a variety of factors, including the presence or absence of prior surgeries, patient compliance, and ability for salvage surgery in the event of contralateral failure.

## 7. Controversies in Patient Selection for Unilateral Neck RT—Oral Tongue Cancer

The management of the clinically node-negative (cN0) contralateral neck for lateralized oral tongue cancer relies on a risk assessment based on imaging and examination. Retrospective surgical series suggests that the risk of occult contralateral neck involvement increases with larger tumors (T3/4) and proximity to the midline [82]. Tumor depth of invasion has been correlated with occult lymph node metastasis [83] and is now part of AJCC 8th edition staging, but this may be confounded by other risk factors associated with thicker tumors [84,85]. When adjuvant RT is indicated, it has traditionally been delivered to both the ipsilateral and contralateral neck [86].

Compared to lateralized tonsil cancer, the data for sparing contralateral neck RT in oral tongue patients are less clear and are summarized in Table 2. Most studies are limited by heterogeneous treatment, poorly described RT, and selection bias. Yet the reported contralateral neck failure rates in the absence of contralateral neck RT in these selected patients with pN0-2a surgical staging are reassuring, ranging from 0–12% [83,84,87,88,89,90,91,92,93,94,95]. While no single risk factor appears to be independently and reliably associated with nodal recurrence, increasing the depth of invasion appears to confer the highest risk when combined with additional risk factors [83]. In a recent multi-institutional cohort of over 1400 cT1-2N0 oral tongue patients, just one additional risk factor in patients with depth of invasion >5 mm doubles the locoregional failure rate to nearly 30% without RT; however, contralateral neck failure rates were not explicitly listed [84]. It is of note that in this study the unilateral neck RT volume still included the contralateral levels IA-B, even if patients had a bilateral neck dissection.

In order to determine the incidence of and factors associated with contralateral neck failure, Udovicich et al. examined the clinical outcomes of patients with oral tongue squamous cell carcinoma treated with a predefined policy contralateral neck radiotherapy between 2007 and 2016 at two Australian centers [96]. Their policy involved performing contralateral neck dissection (ND) when the primary tumor crossed the midline and recommending bilateral elective nodal irradiation (ENI) when the primary tumor was ≤1 cm from the midline or 2 cm from the tip of the tongue [96]. Of the 258 patients in the study, ND was ipsilateral in 169 (66%) and bilateral in 33 (13%), and 55 patients (21%) received ENI to the undissected contralateral neck [96]. There were 51 patients treated with ipsilateral neck dissection and ipsilateral neck RT. With this approach, contralateral neck failure occurred in 19 patients (7%) and was similar by treatment group, but salvage was poor after recurrence (15/19 died of cancer) [96]. Factors associated with increased contralateral neck failures included increasing nodal stage, perineural invasion, extracapsular extension, and depth of invasion ≥6 mm [96]. In the ongoing NRG-HN006 trial comparing sentinel lymph node biopsy with standard neck dissection for patients with early-stage oral cavity cancer, unilateral RT is allowed for patients with AJCC 8th pT1-4, pN0-2b disease, as long as the primary tumors are lateralized (≥1 cm from the midline) [97].

## 8. Future Directions

**Figure 1:**
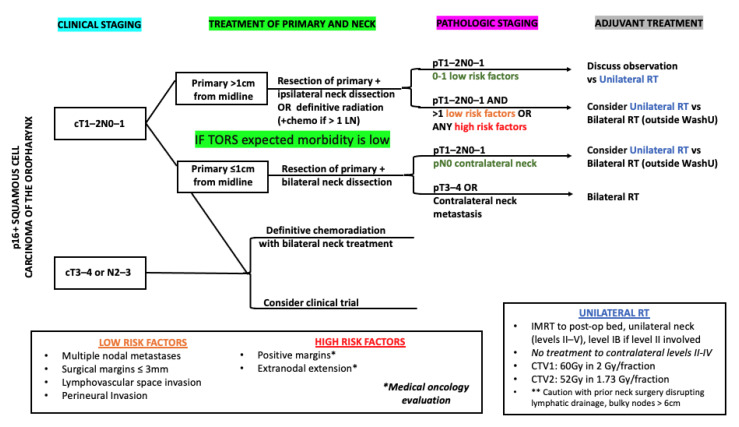
Proposed patient selection (on clinical trial) for unilateral neck RT for patients with p16+ squamous cell carcinoma of the oropharynx staged with PET/CT. * = Patients with tumor with positive margins and/or extranodal extension on pathology should be referred to medical oncology for evaluation for chemotherapy; ** = Unilateral radiation therapy should be delivered with caution in patients with prior neck surgery that disrupts lymphatic drainage.

**Figure 2:**
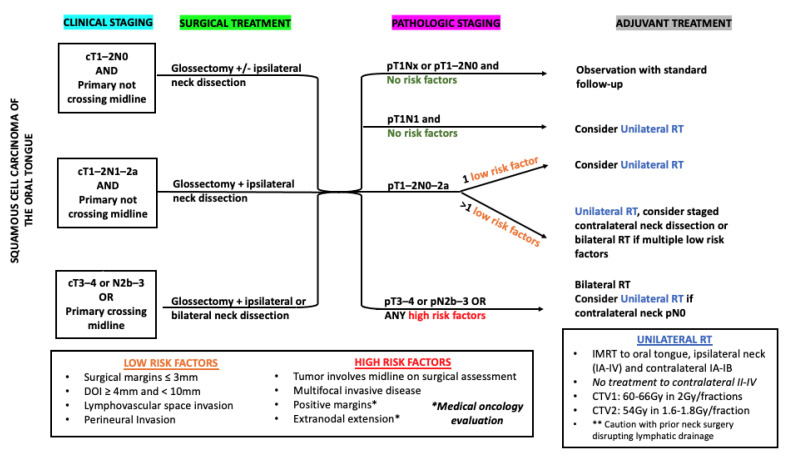
Proposed patient selection (on clinical trial) for unilateral neck RT for patients with squamous cell carcinoma of the oral tongue staged with PET/CT. * = Patients with tumor with positive margins and/or extranodal extension on pathology should be referred to medical oncology for evaluation for chemotherapy; ** = Unilateral radiation therapy should be delivered with caution in patients with prior neck surgery that disrupts lymphatic drainage.

Current trials evaluating the combination of immunotherapy and RT (Table 1) have not changed the standard elective RT volumes. The JAVELIN 100 results [13] were disappointing but may offer an opportunity to accelerate the adoption of novel RT paradigms supported by the preclinical and clinical data presented in this review. One such trial may stratify surgically treated HNSCC by whether the ipsilateral versus bilateral neck RT is delivered with adjuvant immunotherapy. We propose the patient selection criteria for unilateral neck RT for patients with p16+ squamous cell carcinoma of the oropharynx (Figure 1) and oral tongue (Figure 2) as pathways for prospective trial evaluation. Fluorodeoxyglucose (FDG)-positron emission tomography (PET) [99] or novel radiotracers such as fibroblast-activation-protein inhibitors (FAPI)-PET [100] may allow further confidence in ipsilateral neck treatment, if the negative predictive value for contralateral nodal metastasis is sufficient.

We are also learning more about the biomarkers to best select patients that would benefit from immunotherapy. In addition to PD-L1 staining, metrics such as the Immunoscore that assesses CD3/CD8+ tumor-infiltrating lymphocytes [101,102] could improve the identification of patients who are more likely to respond to immunotherapeutics. Treatment-responsive HPV+ oropharyngeal cancers offer an opportunity to both reduce the elective RT volume and the total dose [103]. HN005 is currently enrolling, and arm three consists of a reduced dose RT combined with nivolumab [19]. Patient stratification by ipsilateral versus bilateral radiation in this trial could offer valuable insights regarding the beneficial or detrimental effects of bilateral neck radiotherapy. We hope that the next generation of trials investigating the synergy between immunotherapy and radiotherapy for HNSCC will benefit from the thoughtful consideration of these factors.

## Figures and Tables

**Table 1:** Recent and ongoing phase II-III studies evaluating RT with immunotherapy in head and neck cancer.

Trial Name/NCT Number (Phase)	Disease Site	Control Arm	Experimental Arm	Primary Endpoint	Median Follow-Up	Results/Status	References
JAVELIN 100 NCT02603432 (III)	Locally advanced SCC of H&N	ChemoRT	Addition of avelumab	PFS	14.8 vs. 14.6 months	Hazard Ratio = 1.21 (95% CI: 0.93–1.57), *p* = 0.92	[13]
NCT02684253 (II)	Metastatic SCC of H&N	Nivolumab	Addition of SBRT	ORR	20.2 months	34.5% (95% CI: 19–52.7%) vs. 29.0% (95% CI: 16.1–46.6%), *p* = 0.86	[14]
GORTEC 2015-01 (PembroRad) NCT02707588 (II)	Locally advanced SCC of H&N unfit for cisplatin	RT plus cetuximab	RT plus pembrolizumab	LRC	25 months	59% vs. 60%, *p* = 0.91	[15]
RTOG 3504 NCT02764593 (II)	Intermediate and high-risk SCC of H&N	None	Definitive chemoRT or RT plus cetuximab plus nivolumab	Dose limiting toxicity	Acceptable safety profile		[16]
NCT02641093 (II)	Locally advanced SCC of H&N	None	Neoadjuvant pembrolizumab followed by resection and adjuvant pembrolizumab	Treatment adverse events	Acceptable safety profile		[17]
NRG-HN004 (II)	Locally advanced SCC of H&N unfit for cisplatin	None	RT plus durvalumab	Dose limiting toxicity	Acceptable safety profile		[18]
NRG-HN005 (II)	HPV-positive oropharyngeal cancer, ≤10 pack years of smoking	ChemoRT	RT plus nivolumab	PFS	Actively recruiting		[19]
IMPORT (II) NCT04523883	Intermediate and high-risk SCC of H&N unfit for cisplatin	None	Adjuvant RT plus JS001 (anti-PD-1)	DFS	Actively recruiting		[20]
KEYCHAIN (II) NCT03383094	Advanced/intermediate risk SCC of H&N	Concurrent RT plus cisplatin	Concurrent RT plus pembrolizumab	PFS	Actively recruiting		[21]
NCT03283605 (II)	Oligometastatic SCC of H&N	None	Durvalumab plus tremelimumab plus SBRT	Acute toxicity & PFS	Actively recruiting		[22]
REPORT NCT03317327 (II)	Locally recurrent SCC of H&N	None	Re-irradiation plus nivolumab	Acute Toxicity	Actively recruiting		[23]
NCT03258554 (III)	Locoregionally advanced SCC of H&N unfit for cisplatin	RT plus cetuximab	RT plus durvalumab	Dose limiting toxicity, PFS, OS	Actively recruiting		[24]
KEYSTROKE (II)NCT03546582	Locoregionally recurrent or second primary SCC of H&N	SBRT	SBRT plus pembrolizumab	PFS	Actively recruiting		[25]
NCT03894891	Locoregionally advanced laryngeal and hypopharyngeal cancer	None	Induction docetaxel plus cisplatin followed by concurrent RT plus nivolumab	Laryngectomy-free survival	Actively recruiting		[26]

**Table 2:** Retrospective studies of patterns of failure in early-stage oral cavity cancers.

Study (Year)	Patients	Treatment	Ipsilateral Neck (IN) Failure	Contralateral Neck (CN) Failure	References
Hong Kong (1999)	OT cT1-2N0 (*n* = 28)	WLE alone	13/28 (46%)		[87]
Finland (2006)	OT cT1-2N0 (*n* = 80)	WLE alone (*n* = 34)	7/34 (21%)	0 (0/34) (0%)	[88]
		WLE + IND (*n* = 9) WLE + IND + bRT (*n* = 35) WLE + bRT (*n* = 2)	4/46 (9%)	0 (0/46) (0%)	
Brazil (2006)	OT cT1-2N0 (*n* = 222) FOM cT1-2N0 (*n* = 117)	WLE + IND	77/339 (23%)	3/339 (1%)	[89]
Netherlands (2010)	Lateralized OC/OPX > 1 cm from midline (*n* = 123)	WLE + IND + iRT		pN0 5 yr CN 1% pN1-2a 5 yr CN 12% pN2b 5 yr CN 27%	[90]
MSKCC/Princ. Margaret (2013)	OT cT1-2N0 (*n* = 164)	WLE + IND	18/164 (11%)	11/164 (7%)	[83]
Germany (2017)	OT cT1-2N0 (*n* = 150); didn’t cross midline	WLE + IND (*n* = 105), *n* = 27 with RT*	11/105 (10%)	3/105 (3%)	[91]
		WLE + BND (*n* = 45), *n* = 11 with RT*	3/45 (7%)	1/45 (2%)	
Italian (2017)	OC, >95% cT1-2N0 (*n* = 231)	WLE alone (*n* = 130)	39/130 (30%), laterality not specified		[92]
		WLE + IND; BND if crosses midline, (*n* = 101), *n* = 20 with RT*	36/101 (37%), laterality not specified		
Japanese (2017)	OC: 39% OT, 65% cT2, 95% pN0-1 (*n* = 188)	WLE + IND (*n* = 178) WLE + BND (*n* = 10)	3/188 (2%)	4/188 (2%)	[98]
U Florida (2019)	OT + FOM (*n* = 32), 75% <5 mm margins, 38% PNI+, 68% DOI > 5 mm; none crossed midline	WLE + IND (*n* = 21) WLE + BND (*n* = 7) iRT (*n* = 19)		0/47 (0%)	[93]
Multi-inst. (2019)	OC, cT1-2N0 (*n* = 1409)	WLE alone (*n* = 407) WLE + ND (*n* = 1002) *n* = 697 without RT*	No other adverse features and no RT subgroup (*n* = 625) DOI < 5 mm 5 yr failure 14% DOI 5–10 mm 5 yr failure 15% DOI > 10 mm 5 yr failure 17%		[84]
Taiwan (2020)	OT, cT1-2N0 (*n* = 199)	WLE alone (*n* = 96) WLE + IND (*n* = 103)	5 yr LRFFS 77% vs. 67% with any DOI >5 mm, PNI, or poorly diff histology		[94]
China (2020)	OT, cT1-2N0 (*n* = 219)	WLE + IND (*n* = 169)	18/169 (11%)	5/169 (3%)	[95]
		WLE alone (PET cN0, *n* = 50); RT* given in about 20% of pts in each group	8/50 (16%)	3/50 (6%)	
Australia (2021)	OT (*n* = 258)	WLE (*n* = 44)		3-year CN failure: 9% for no CN treatment	[96]
		WLE with IND or iRT (*n* = 126)			
		WLE with BND or bRT (*n* = 88)		7% for BND alone 8% for bRT alone 8% for BND/bRT	

OT—Oral Tongue; OC—Oral cavity; OPX—Oropharynx; FOM—Floor of mouth; WLE—Wide local excision; IN—Ipsilateral neck; CN—Contralateral neck; IND—Ipsilateral neck dissection; BND—Bilateral neck dissection; iRT—Ipsilateral neck RT only; bRT—Bilateral neck RT; RT*—RT laterality not specified; LRFFS—Locoregional failure free survival.
